# Investigation of the potential role of TGR5 in pancreatic cancer by a comprehensive molecular experiments and the liquid chromatography mass spectrometry (LC–MS) based metabolomics

**DOI:** 10.1007/s12672-022-00504-2

**Published:** 2022-06-11

**Authors:** Yangyang Lei, Guoping Li, Jianke Li, Shanshan Gao, Ming Lei, Gaoquan Gong, Changyu Li, Yi Chen, Chenggang Wang, Xiaolin Wang

**Affiliations:** 1 Department of Interventional Radiology, Zhongshan Hospital, Fudan University, Shanghai, 200032 China; 2 Shanghai Institute of Medical Imaging, Shanghai, 200032 China; 3 National Clinical Research Center for Interventional Medicine, Shanghai, 200032 China; 4 Department of Liver Surgery and Transplantation, Liver Cancer Institute and Zhongshan Hospital, Fudan University, Shanghai, 200032 China

**Keywords:** TGR5, Pancreatic cancer, Liquid chromatography mass spectrometry, SBI-115, Mitochondria

## Abstract

**Background:**

Takeda G protein receptor 5 (TGR5) is widely recognized as a potential drug target for the treatment of metabolic diseases. TGR5 is not only a metabolic regulator, but also has a potential role that participating in developing and progressing of gastrointestinal cancer. We aimed to investigate the potential role of TGR5 in pancreatic cancer by utilizing molecular experiments and the liquid chromatography mass spectrometry (LC–MS) based metabolomics.

**Methods:**

Herein, we assessed pancreatic cancer proliferation, migration and invasion in response to TGR5 antagonist SBI-115 in vitro experiments. Cell death was examined by using TUNEL assay on agarose-embedded sections. Then we investigated the effects of TGR5 on PANC-1 and BXPC3 cells via transmission electron microscopy (TEM). Moreover, LC–MS-based metabolomics was performed to explore the potential underlying mechanisms of TGR5 in pancreatic cancer. The correlations between TGR5 and the metabolism-related genes were further analysed by GEPIA 2 database.

**Results:**

We found the proliferation capacities were decreased significantly in PANC-1 and BXPC3 cells after the treatment of SBI-115 for 48 h. The results of TUNEL assay showed that antagonism of TGR5 by SBI-115 had a remarkable effect on inducing cell death. Analysis of TEM demonstrated that SBI-115 treatment could impair the morphology of mitochondria in most PANC-1 and BXPC3 cells. The LC–MS-based analyses revealed that antagonism of TGR5 could alter the metabolic profiles of PANC-1 cells in vitro*.* Moreover, TGR5 was associated with some metabolism-related genes in pancreatic cancer.

**Conclusion:**

Our data suggests that antagonism of TGR5 may suppress cell proliferation and induce apoptosis in pancreatic cancer cells. TGR5 may affect the metabolism of pancreatic cancer, and TGR5 would be an attractive target for pancreatic cancer treatment.

**Supplementary Information:**

The online version contains supplementary material available at 10.1007/s12672-022-00504-2.

## Introduction

Bile acids, synthesized from cholesterol in the liver, are metabolized by enzymes produced by intestinal bacteria and are also essential in the maintenance of metabolic homeostasis, insulin sensitivity and innate immunity [1]. Bile acids are highly cytotoxic and regarded as cancer promoters in esophagus, stomach, pancreas, liver and colon. High levels of bile acids in the body predict an increased incidence of tumors originating from these organs [2]. TGR5 is a G protein-coupled bile acid receptor responsive to energy homeostasis, glucose metabolism and bile acid homeostasis [3]. Increasing evidence points to an essential role of TGR5 in responding to bile acid within enterohepatic organs, such as liver, gallbladder and intestine [4]. TGR5 can be activated by bile acids and regulates various signaling pathways such as nuclear factor κB (NF-κB), AKT as well as extracellular signal-regulated kinases (ERK) [5–8]. Due to the important roles in different signaling pathways, TGR5 becomes a promising target in many diseases. Its agonists may have the potential in the treatment of metabolic and digestive disorders [9, 10]. Moreover, TGR5 is of clinical significance based on the role in carcinogenesis, including gastric cancer, esophageal cancer and hepatocellular carcinoma (HCC) [11–13]. Nonetheless, the effect of TGR5 in pancreatic cancer remains controversial. It has been found that TGR5 is expressed at pancreatic acinar cells and acts as an important mediator in bile acid-induced pancreatitis. Previous study also revealed that TGR5 expression was increased in pancreatic cancer tissues compared with the adjacent normal tissues. Meanwhile, TGR5 may be an independent predictor of overall survival in pancreatic cancer patients [14, 15]. Thus, the further deeper research remains of great importance. Therefore, a novel small molecule TGR5 antagonist SBI-115 was utilized to assess the effect of antagonizing TGR5 on pancreatic cancer cells [16].

## Material and method

### Cell culture

The human pancreatic cancer cell lines PANC-1, ASPC1, BXPC3, CFPAC-1 and MIA PaCa-2 were kindly provided by Department of General Surgery, Zhongshan Hospital, Fudan University and kept as previously reported [17, 18]. All cell lines have been identified and confirmed to be free of mycoplasma contamination before the experiments. Cells were cultured in 5% CO_2_ and 95% air incubator, with the temperature at 37 ℃.

### Western blot analysis

Total proteins of above cell lines were lysed with RIPA buffer (Biosharp, BL651A) supplemented with 1% PMSF (Beyotime, ST506), and then mixed with loading buffer. After boiling for 10 min at 100 ℃, equal amounts of protein were separated by 10% SDS-PAGE (Beyotime) and then transferred to polyvinylidene fluoride (PVDF) membranes (Millipore, Billerica, MA, USA). For blocking the nonspecific binding, the membranes were incubated with 5% skim milk at room temperature for 2 h and then probed with anti-TGR5 antibody (1:1000, Abcam, ab72608) and anti-β-actin (1:2000, Cell Signaling, Rabbit mAb, 4970) overnight at 4℃. After being rinsed with TBS-T (TBS containing 0.05% Tween-20), the PVDF membranes were incubated with HRP-conjugated secondary antibody (1:5000, Abcam, ab6721) for 2 h at room temperature. Afterwards, the proteins were detected by utilizing an enhanced chemiluminescence kit (New Cell & Molecular Biotech Co., Ltd, Suzhou, Jiangsu, China) and β-actin was used as an internal control. To determine the relative quantity of each protein band, ratios of TGR5 proteins/ β-actin were performed using ImageJ software.

### CCK8 assay

In order to find the suitable antagonistic concentrations of each cell line in response to SBI-115, the different final concentrations of SBI-115 (TGR5 antagonist; MCE, HY-111534) were set as 20, 10, 5 and 1 μM by utilizing the CCK8 assay, and an equal amount of dimethyl sulfoxide (DMSO) was added into the control group. The pancreatic cancer cell lines were collected and seeded into 96-well plates with a density of 3 × 10^4^ cells/ml, 100 μl per well for 24 h. Then SBI-115 was added into per well with different final concentrations. Afterwards, the cells were incubated with 10 μl CCK8 solution (Dojindo, Kumamoto, Japan) per well for 2 h at 37℃ at different detection time points. The optical density (OD) was detected with at a wavelength of 450 nm.

### Clonal formation assay

The cells were seeded into the six-well plate at a density of 1000 cells per well in triplicate and followed by incubating in complete culture medium at 37℃ for 15d. The cells were washed with PBS and then fixed with 4% paraformaldehyde for 30 min. Afterwards, 0.1% (w/v) crystal violet was utilized to stain the cells. The number of colonies was counted by using ImageJ software. All assays were performed in triplicate.

### Wound healing assay

The wound healing assay was performed to assess the cell migration capacity. Briefly, the cells were collected and seeded into 6-well plates. When the cells reached 100% confluence, a 200-µl plastic pipette tip was used to make a scratch on the cell monolayer followed by 3 washes in PBS. After removing the cell debris, the serum-free DMEM was added into 6-well plates. The wounds were recorded at 0 h and 48 h. The extent of wound closure was analyzed by using ImageJ software and all assays were performed in triplicate.

### Transwell cell migration and invasion assays

The 12-well transwell chamber (8 µm pore size; Corning, NY, USA) was applied to analyze the migratory and invasive abilities of pancreatic cancer cells. For the transwell cell migration assay, 2 × 10^5^ pancreatic cancer cells in 200 µl serum-free DMEM were directly seeded into the upper chamber, while 800 µl DMEM with 10% FBS was added to the lower chamber. After 24 h incubation, the migrating cells were fixed with 4% paraformaldehyde and stained with crystal violet. For the transwell cell invasion assay, the 12-well transwell chamber was coated with 50 µl of 1:8 mixture of Matrigel (BD Biosciences) and serum-free DMEM for 2 h at 37 ℃. Then the 4 × 10^5^/ml pancreatic cancer cells were seeded into the upper chamber and 800 µl DMEM containing 10% FBS was added to the lower chamber. After 24 h, the cell was fixed and stained followed by imaging at 100 × magnification under an inverted light microscope. The number of cells that migrated and invaded through the chamber filter was counted by the ImageJ software.

### TUNEL assay

To detect the dying cells, the terminal deoxynucleotidyl transferase-mediated uridine 5′-triphosphate-biotin nick-end labeling (TUNEL) assay was performed. Briefly, the cells were collected from the six-well plate and fixed with paraformaldehyde. Afterwards, these cells were gently embedded in the low-melting-point agarose after centrifugation at 1600 rpm for 5 min. Then the agarose-embedded sections were incubated in 0.1% of Triton X at room temperature for 10 min after dehydrating in different gradients of alcohol. Then the TUNEL kit (Sigma, USA) was used for the estimation of apoptosis.

### Transmission electron microscopy

The cells in SBI-115-treated group and DMSO control group were collected after centrifuge. Then TEM fixative was added and let the precipitation re-suspended in the fixative. After a graded ethanol series, the fixed cells were dehydrated and embedded in the pure EMBed 812, and then kept in 37 ℃ overnight. The TEM was performed and took pictures.

### Non-targeted LC–MS metabolic profiling

After treatment with SBI-115 or DMSO (control group) in PANC-1 cells for 48 h, the culture supernatants were collected from SBI-115-treated cells (n = 6) and DMSO-treated cells (n = 6) for LC–MS based non-targeted metabolomics analyses. The detailed protocol was as follows according to Shanghai Lu-Ming Biotech Co., Ltd. 1000 μl supernatants from each sample were added to an 1.5 ml microtube and then dried in a freeze drier. L-2-chlorophenylalanine (0.3 mg/mL) dissolved in methanol as internal standard and the mixture of methanol and water (1/4, vol/vol) were added to each sample. After being extracted by ultrasonic in ice-water and placed at − 20℃ for 2 h, the samples were centrifuged at 13,000 rpm speed for 10 min. The supernatants were collected using crystal syringes and transferred to LC vials for LC–MS analysis. After analyzed by the Nexera UPLC system (Shimadzu Corporation, Japan), the original LC–MS data was processed by software Progenesis QI V2.3 (Nonlinear, Dynamics, Newcastle, UK) for baseline filtering, peak identification, integral, retention time correction, peak alignment, and normalization. The principal component analysis (PCA) and Orthogonal Partial Least-Squares-Discriminant Analysis (OPLS-DA) were utilized to distinguish the metabolites that differed between groups. To prevent overfitting, sevenfold cross-validation and 200 Response Permutation Testing (RPT) were used to evaluate the quality of the model. Variable Importance of Projection (VIP) values obtained from the OPLS-DA model were used to rank the overall contribution of each variable to group discrimination. The fold change (FC), VIP value and P value were used to screen the differential metabolites as follows: VIP ≥ 1 from the cross-validated OPLS-DA models, P value < 0.05 and |log2FC|> 0.25.

### The correlation analyses between TGR5 and the metabolism-related genes by GEPIA 2

To further validate the relationships between TGR5 expression level and the metabolic pathways in pancreatic cancer, the metabolism-related genes were chosen for correlation analyses by GEPIA 2 (http://gepia2.cancer-pku.cn/). GEPIA 2 is an enhanced web server which allows users to analyze gene expression profiles based on tumor and normal samples from the TCGA and the GTEx databases [19]. Gene expression correlation analyses were performed by the Spearman method using the "Correlation" module of GEPIA 2. The “PAAD Tumor”, “PAAD Normal” and “Pancreas” were selected for analysis. The strength of correlation was calculated as (R). TGR5 was represented on the x-axis, and other genes were represented on the y-axis.

### Statistical analysis

Statistical analysis was performed using SPSS 23.0 software (SPSS, Inc., Chicago, IL, USA). The continuous values of experiments were expressed as means ± standard deviation (SD). The differences were analyzed by using Student's t-test when comparing only two groups. The strength of the correlation was determined as follows: very weak (0.00–0.19), weak (0.20–0.39), moderate (0.40–0.59), strong (0.60–0.79), and very strong (0.80–1.0) [20]. P < 0.05.*P < 0.05, **P < 0.01 or ***P < 0.001 were considered statistically significant.

## Results

### SBI-115 can antagonize the expression of TGR5 at protein level in PANC-1 and BXPC3 cells

**Fig. 1:**
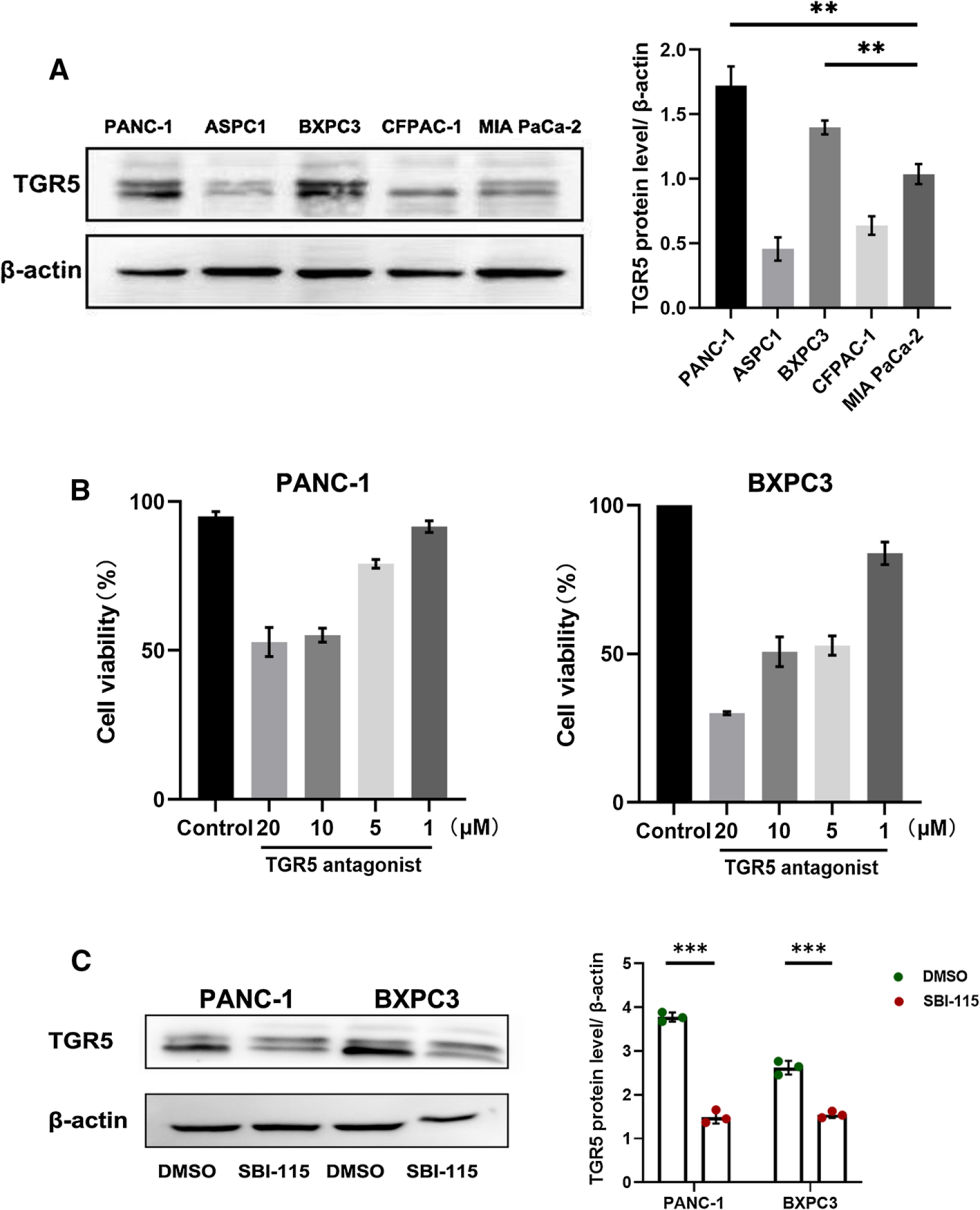
SBI-115 can antagonize the expression of TGR5 in pancreatic cancer cells. **A** The protein levels of TGR5 in five human pancreatic cancer lines (PANC-1, ASPC1, BXPC3, CFPAC-1, MIA PaCa-2); **B** The effects of different concentrations of SBI-115 on the cell viabilities of PANC-1 and BXPC3 cell lines by using CCK-8; **C** The result of Western blot showed that TGR5 expressions in SBI-115-treated PANC-1 and BXPC3 groups were weakened compared with that control groups. **P < 0.01, ***P < 0.001

Human pancreatic cancer cell lines have different characteristics and remain the biological complexity, such as DNA fingerprinting, functional profiles, et al. [21, 22]. To explore the potential role of TGR5 in pancreatic cancer, its protein level was first examined by western blot in a panel of five human pancreatic cancer (PANC-1, ASPC1, BXPC3, CFPAC-1, MIA PaCa-2). Western blot analyses showed that TGR5 protein levels were upregulated both in PANC-1 and BXPC3 cell lines compared with those in ASPC1, CFPAC-1 and MIA PaCa-2 pancreatic cancer cells (Fig. 1A) (P < 0.01). Based on these results, we performed the follow-up TGR5 antagonism studies with the PANC-1 and BXPC3 cells (TGR5-high) instead of the ASPC1, CFPAC-1 and MIA PaCa-2 cells (TGR5-low). Then the suitable antagonistic concentration values of SBI-115 on PANC-1 and BXPC3 cells were explored by CCK8 assay. After 48 h of SBI-115 treatment, the concentrations resulted in about 50% percent death of PANC-1 and BXPC3 cells were chosen for the subsequent study. The results indicated that SBI-115 exhibited significant inhibitory activities against the PANC-1 and BXPC3 cell lines at 10 μM and 5 μM, respectively (Fig. 1B). In order to validate whether SBI-115 antagonized the expression of TGR5, the western blot assay was applied to detect the protein level of TGR5. We found that TGR5 expressions in PANC-1 and BXPC3 cells were weakened within 48 h after the treatment of SBI-115 at 10 μM and 5 μM, respectively (Fig. 1C) (P < 0.001). These results indicated that SBI-115 can antagonize the expression of TGR5 at protein level in pancreatic cancer cell lines.

### Pancreatic cell proliferation is inhibited while antagonizing TGR5

**Fig. 2:**
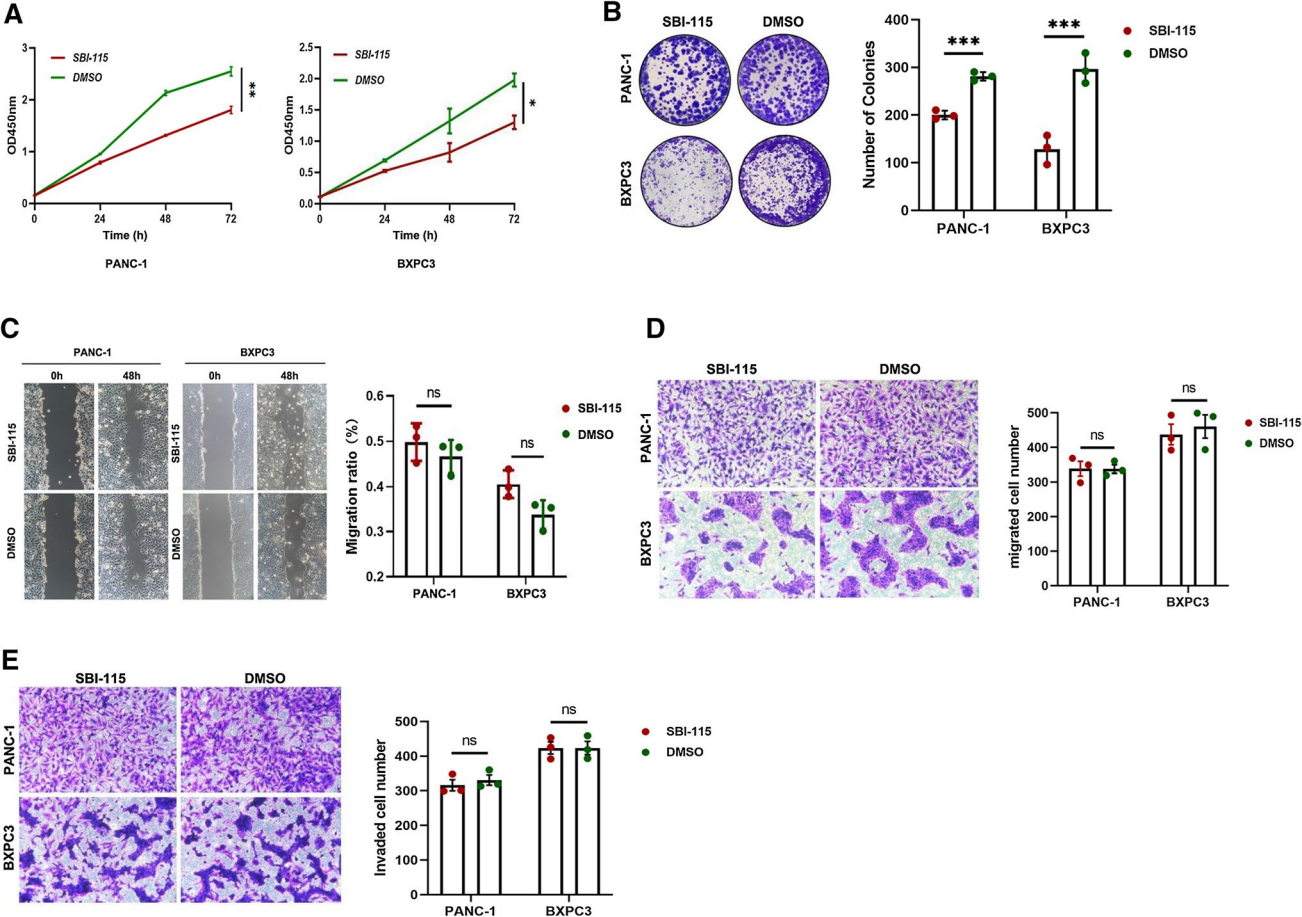
Antagonism of TGR5 by SBI-115 suppressed cell proliferation, but had no effects on cell migration and invasion in PANC-1 and BXPC3 cells. **A** The effects of SBI-115 on cell proliferation by CCK-8 in PANC-1 and BXPC3 cells; **B** The effects of SBI-115 on cell proliferation by using colony formation assays in PANC-1 and BXPC3 cells; **C** The effects of SBI-115 on cell migratory abilities by scratch wound healing assays in PANC-1 and BXPC3 cells; **D** The effects of SBI-115 on cell migratory abilities by transwell assay in PANC-1 and BXPC3 cells; **E** The effects of SBI-115 on cell invasive capacities by transwell assay in PANC-1 and BXPC3 cells. *P < 0.5, **P < 0.01, ***P < 0.001, *ns* no significant

The results of cell proliferation by CCK-8 and colony formation showed that the proliferation abilities of SBI-115-treated PANC-1 and BXPC3 cells were decreased compared with the control groups (Fig. 2A, B). However, the results of scratch wound healing assays and transwell cell migration assays indicated that antagonizing TGR5 had no effect on the pancreatic cancer cell migratory capacities (Fig. 2C, D). In addition, the results of transwell cell invasion assays also indicated that antagonizing TGR5 had no effect on the pancreatic cancer cell invasive capacities (Fig. 2E).

### Antagonizing TGR5 induces pancreatic cancer cell apoptosis in vitro

**Fig. 3:**
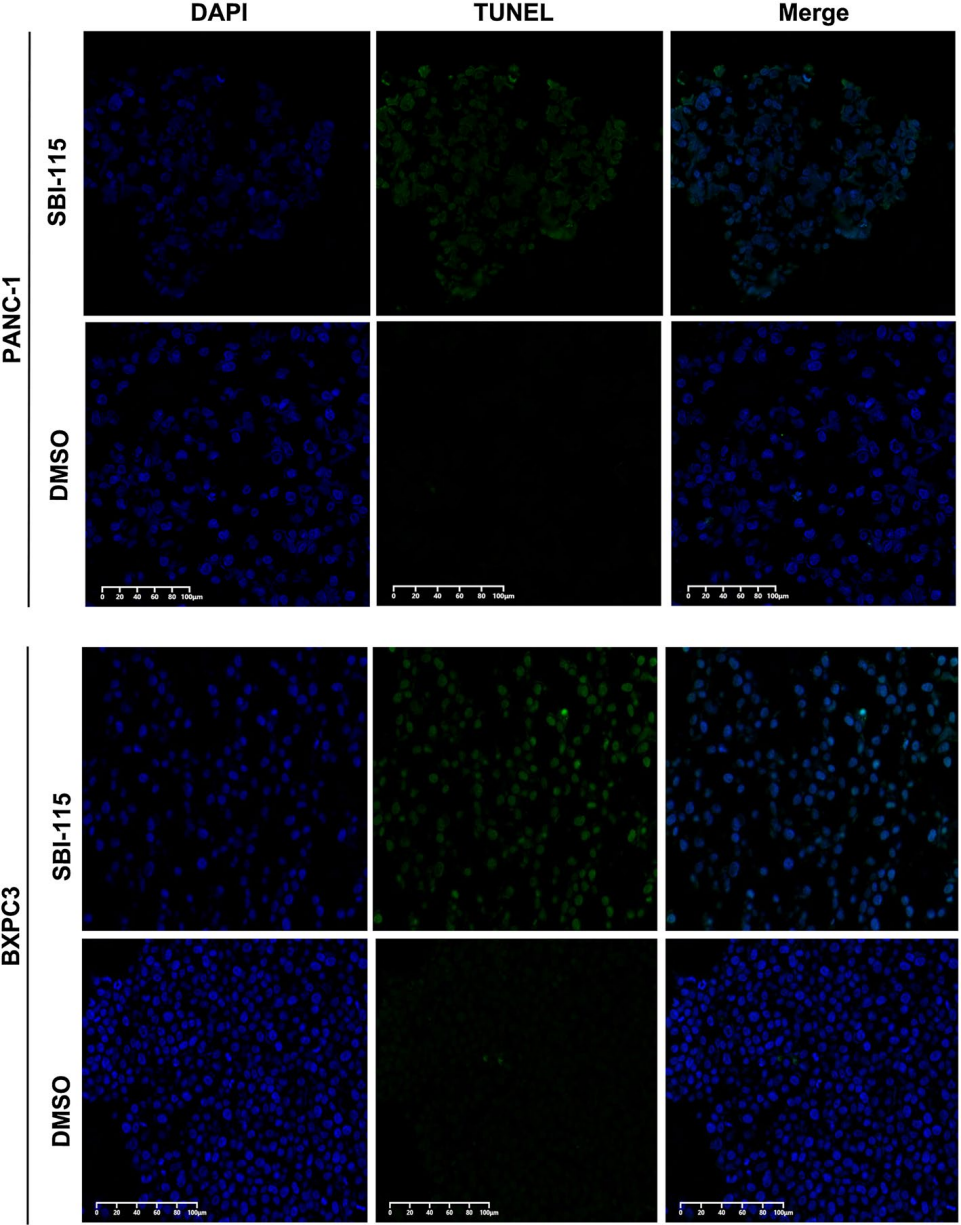
Antagonizing TGR5 induced pancreatic cancer cell apoptosis in vitro by using TUNEL assay. (green, TUNEL-positive; blue, DAPI). Scale bar:100 μm

To further explore the oncogenic role of TGR5 in pancreatic cancer, TUNEL assay was used for apoptosis detection.The results indicated that the proportions of TUNEL-positive PANC-1 and BXPC3 cells in SBI-115-treated-groups were higher than those in control groups (Fig. 3).

### The cell morphologies and ultrastructural changes induced by SBI-115 in vitro

**Fig. 4:**
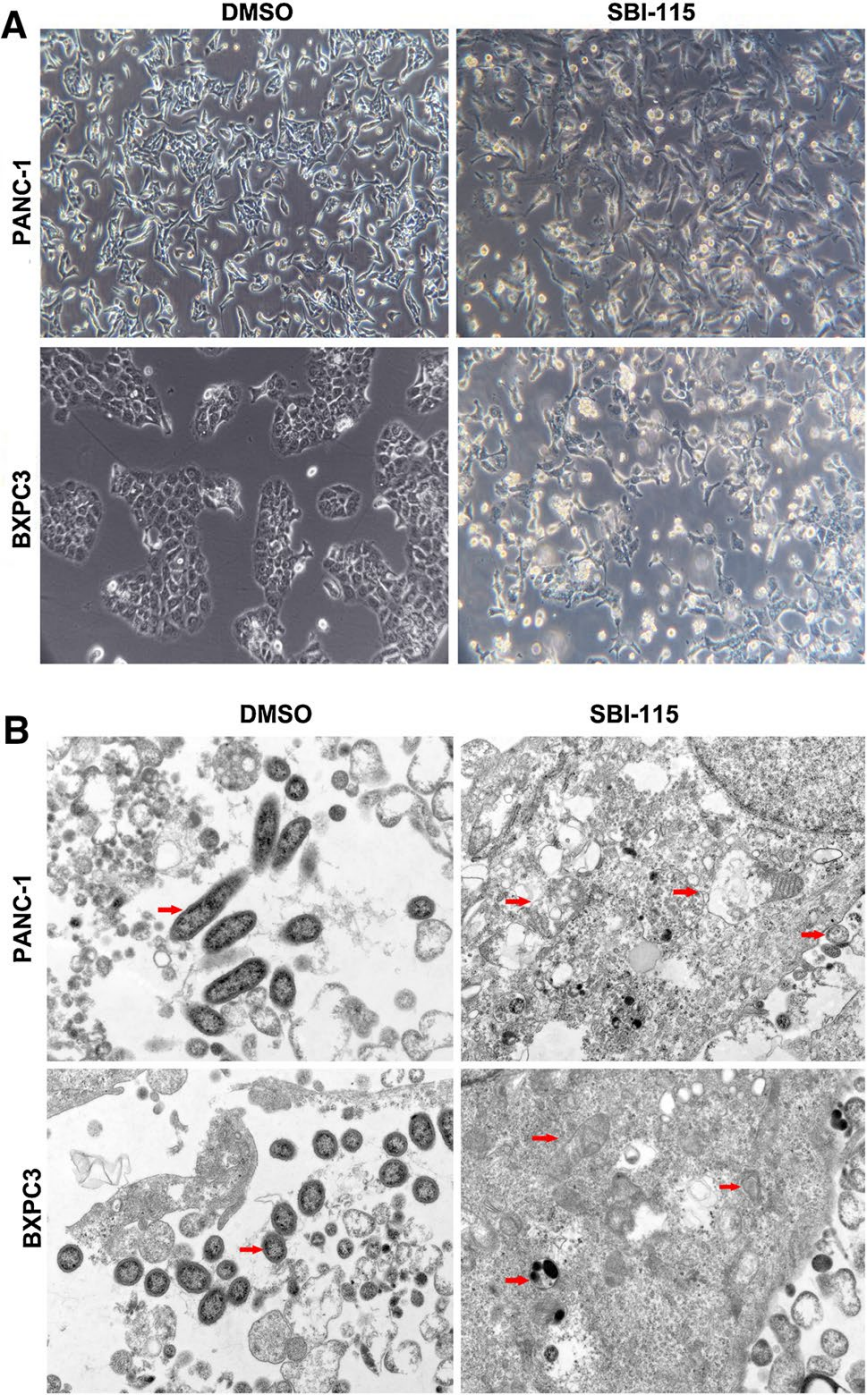
The cell morphologies and ultrastructural changes induced by SBI-115 in vitro. **A** The cellular morphologies of PANC-1 and BXPC3 cells were observed by using the light inverted microscope (100 ×); **B** The ultrastructural changes in PANC-1 and BXPC3 cells were observed by transmission electron microscope (1 μm). The red arrows indicated the mitochondria

For further research, we treated cells with SBI-115 or DMSO at an initial density of 3 × 10^4^ cells/ml for 48 h in complete culture medium, then the clear visualization of cell morphologies and ultrastructural changes were observed under inverted microscope and transmission electron microscope, respectively. In the control group, human PANC-1 and BXPC3 cells exhibited full body, clear border and close arrangement. Meanwhile, PANC-1 and BXPC3 cells treated with SBI-115 showed unregulated-shaped body and more visible vacuoles (Fig. 4A). Furthermore, the transmission electron microscopy was used to determine the ultrastructural changes in SBI-115-treated cells. It was shown that the mitochondria in SBI-115-treated cells exhibited decreased mitochondrial cristae, swollen-shaped body and ruptured mitochondrial membranes while the mitochondria in DMSO control groups were linear with an integral structure (Fig. 4B).

### Antagonism of TGR5 alters the metabolic profiles in PANC-1 cells

**Fig. 5:**
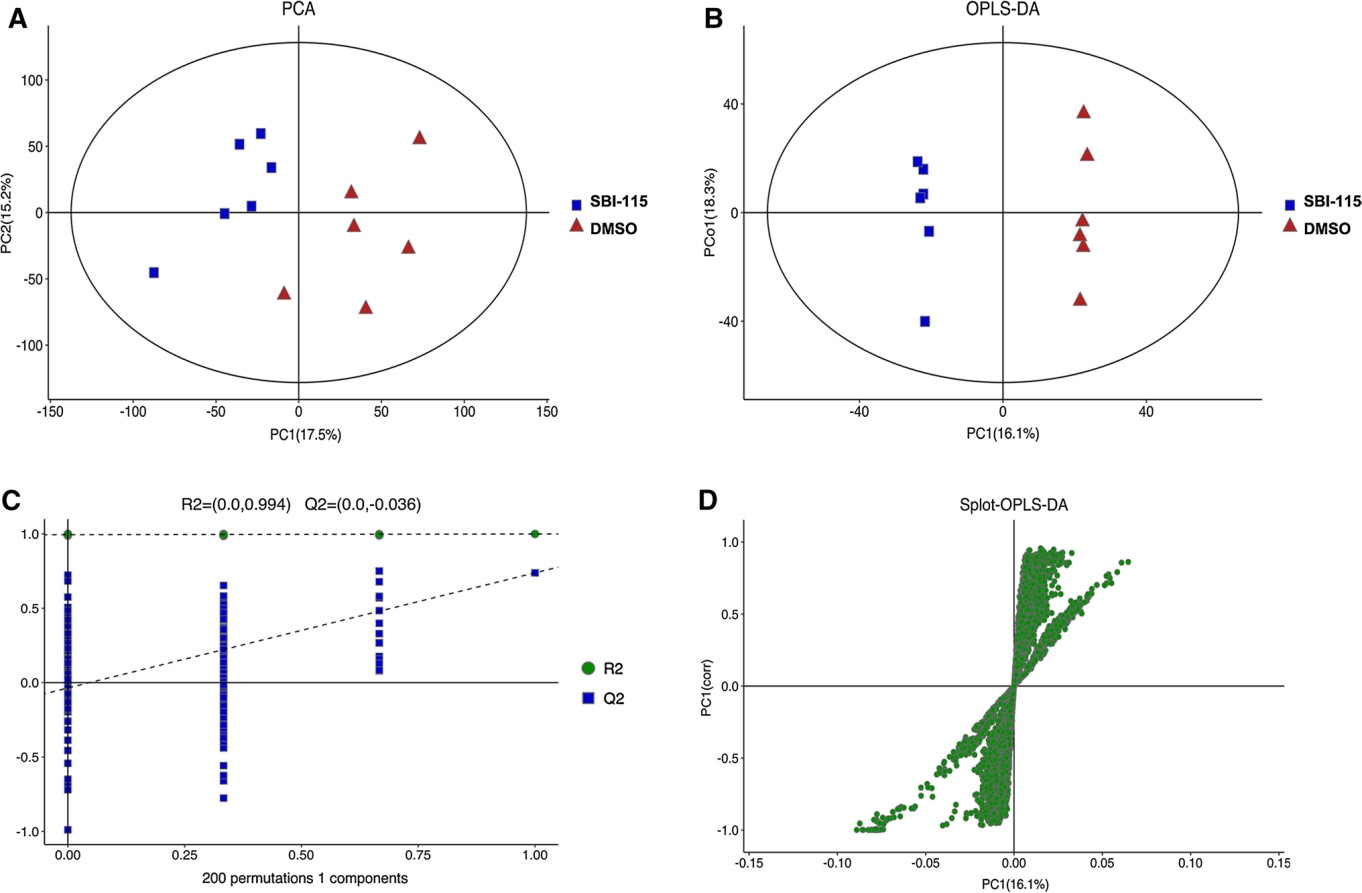
The LC–MS based non-targeted metabolomics analyses in SBI-115-treated group and DMSO-treated group. **A**, **B** The PCA score plot and OPLS-DA score plot showed the differences in metabolic components were significant between the SBI-115-treated group and DMSO-treated group; **C** The permutation test (200 times) of the OPLS-DA model in the SBI-115-treated group and DMSO-treated group; **D** The S-plot derived from OPLS-DA demonstrated the accumulated differential metabolites in the SBI-115-treated-group and DMSO-treated group. *LC–MS* liquid chromatography-mass spectrometry, *DMSO* dimethyl sulfoxide, *PCA* principal component analysis, *OPLS-DA* Orthogonal Partial Least-Squares-Discriminant Analysis

**Fig. 6:**
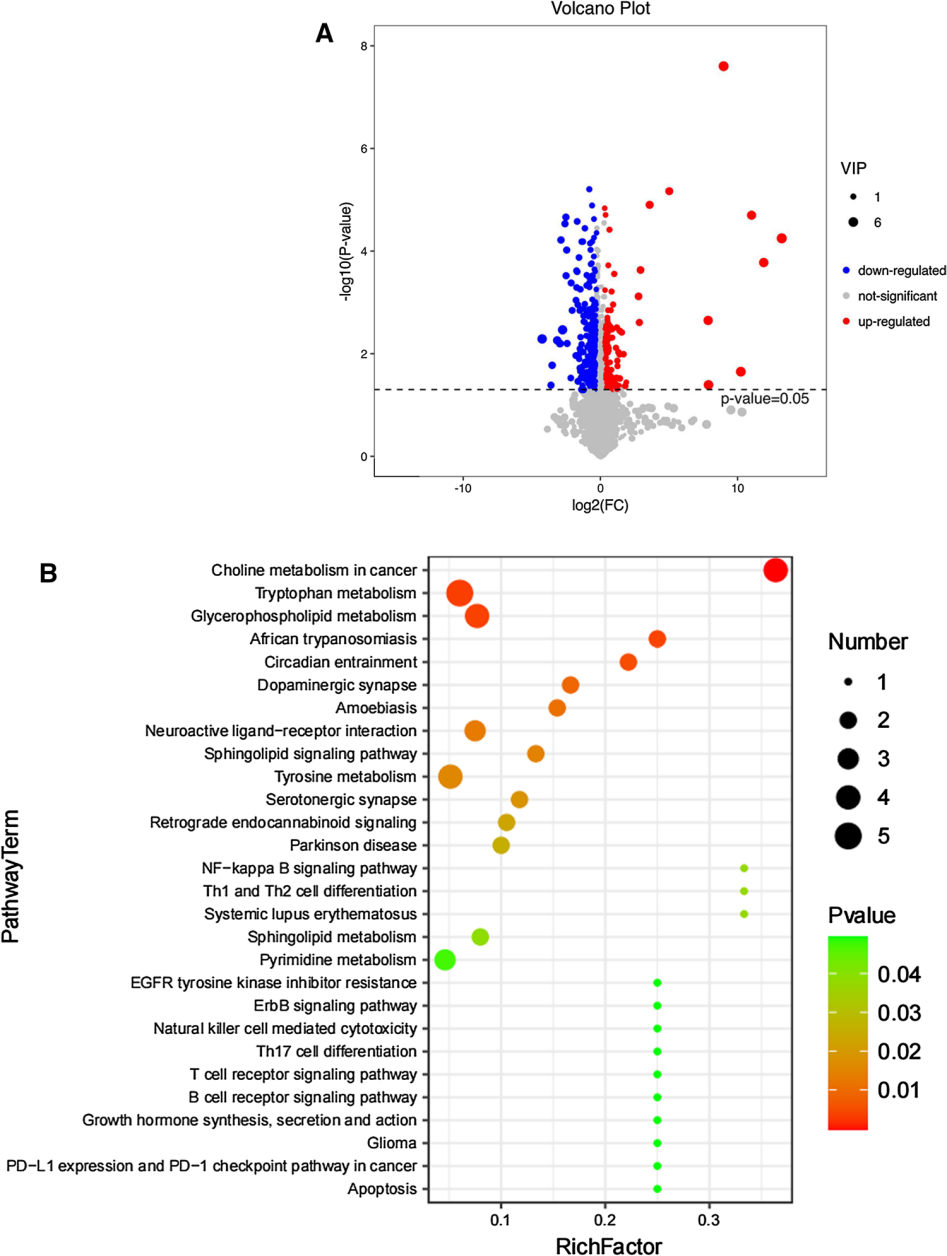
Volcano map of differential metabolites and KEGG pathway enrichment analysis based on the LC–MS metabolomics assay. **A** The volcano map displayed 122 up-regulated metabolites and 245 down-regulated metabolites in SBI-115-treated-group when compared with the DMSO-treated group. The blue and red dots represented the significantly down-regulated and up-regulated metabolites in SBI-115-treated-group, respectively; **B** The KEGG pathway enrichment analysis was expressed as a bubble diagram. The size of the each bubble indicated the number of metabolites. The color of bubbles represented different P values. *KEGG* Kyoto Encyclopedia of Genes and Genomes, *LC–MS* liquid chromatography–mass spectrometry, *DMSO* dimethyl sulfoxide

The LC–MS based non-targeted metabolomics analyses were performed in culture supernatants to compare the metabolic profiling changes between the SBI-115-treated-group and the DMSO-treated group in PANC-1 cells. The PCA and OPLS-DA score plots indicated the great differences between these two groups and the distributions were relative clustered (Fig. 5A, B). Moreover, the values of R2 = 0.994 and Q2 = − 0.036 demonstrated the stable and reliable analytical platform in the OPLS-DA model (Fig. 5C). The S-plot derived from OPLS-DA suggested the accumulated differential metabolites in SBI-115-treated-group and the DMSO-treated group (Fig. 5D). Comparing the metabolites of SBI-115-treated-group versus those of DMSO-treated group, a total of 367 differential metabolites were screened. Among them, 122 differential metabolites were significantly up-regulated while 245 differential metabolites were down-regulated in SBI-115-treated-group when compared with the DMSO-treated group. As shown in volcano map, the blue and red dots represented the significantly down-regulated and up-regulated metabolites in SBI-115-treated-group, respectively (Fig. 6A). Moreover, KEGG pathway enrichment analyses showed the altered metabolites were significantly related to (1) choline metabolism in cancer; (2) tryptophan metabolism; (3) glycerophospholipid metabolism, et al. (Fig. 6B) (Fig. S1A–C). The altered metabolites in above KEGG pathways were represented in Table 1. Fold change (FC) values represented the ratios of average amount of metabolites in SBI-115-treated groups versus DMSO-treated groups.

**Table 1:** The differential metabolites in choline metabolism, tryptophan metabolism and glycerophospholipid metabolism between SBI-115-treated-group and DMSO-treated group

Metabolites	FC	log2(FC)	Pathway
DG(18:1(9Z)/20:1(11Z)/0:0)	0.55513	− 0.849090462	Choline metabolism in cancer
Glycerophosphocholine	0.70270	− 0.509014878	Choline metabolism in cancer|Glycerophospholipid metabolism
LysoPC(18:3(6Z,9Z,12Z))	0.64834	− 0.625187709	Choline metabolism in cancer|Glycerophospholipid metabolism
LysoPC(20:4(5Z,8Z,11Z,14Z))	0.74375	− 0.427108957	Choline metabolism in cancer|Glycerophospholipid metabolism
LysoPC(18:0)	0.69702	− 0.520734807	Choline metabolism in cancer|Glycerophospholipid metabolism
LysoPC(18:1(11Z))	0.77527	− 0.367236082	Choline metabolism in cancer|Glycerophospholipid metabolism
LysoPC(22:5(7Z,10Z,13Z,16Z,19Z))	0.73401	− 0.446119727	Choline metabolism in cancer|Glycerophospholipid metabolism
LysoPC(20:4(8Z,11Z,14Z,17Z))	0.68483	− 0.546189019	Choline metabolism in cancer|Glycerophospholipid metabolism
LysoPC(20:5(5Z,8Z,11Z,14Z,17Z))	0.61572	− 0.699648231	Choline metabolism in cancer|Glycerophospholipid metabolism
PC(20:4(8Z,11Z,14Z,17Z)/0:0)	0.45404	− 1.139117558	Choline metabolism in cancer|Glycerophospholipid metabolism
PC(22:5(4Z,7Z,10Z,13Z,16Z)/0:0)	0.78186	− 0.355018519	Choline metabolism in cancer|Glycerophospholipid metabolism
PC(14:0/20:2(11Z,14Z))	6.87027	2.78036695	Choline metabolism in cancer|Glycerophospholipid metabolism
PC(14:0/20:0)	32.44872	5.020089663	Choline metabolism in cancer|Glycerophospholipid metabolism
PS(18:0/18:1(9Z))	0.41814	− 1.25794216	Glycerophospholipid metabolism
3-Indoleacetonitrile	1.52983	0.613368323	Tryptophan metabolism
3-Methylindole	0.71455	− 0.484888514	Tryptophan metabolism
6-Hydroxymelatonin	0.70671	− 0.50080222	Tryptophan metabolism
Melatonin	0.49556	− 1.012876024	Tryptophan metabolism
L-Tryptophan	1.44946	0.535518232	Tryptophan metabolism

### Relationships between TGR5 and the metabolism-related genes in pancreatic cancer

**Fig. 7:**
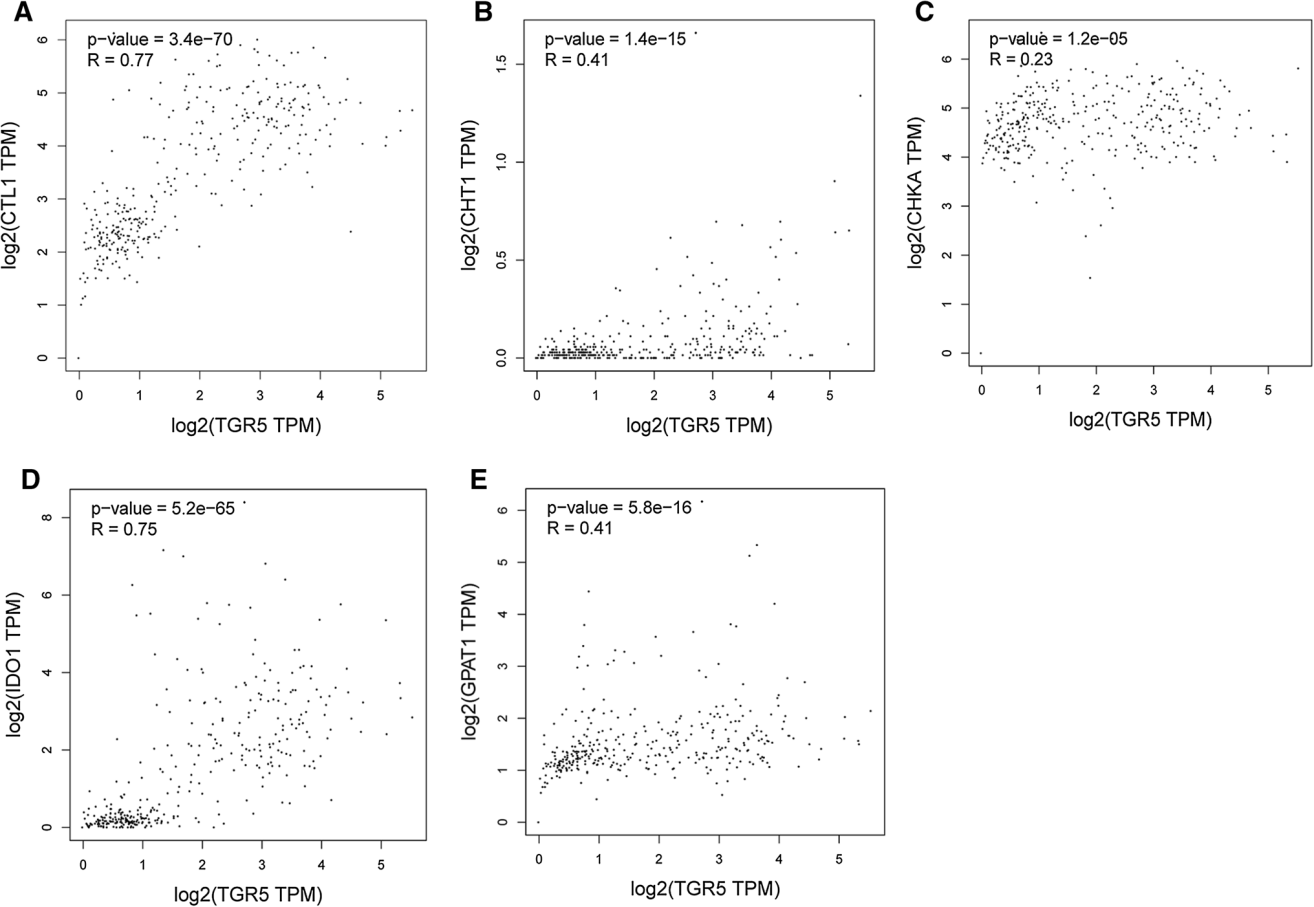
The correlation analyses between TGR5 and the metabolism-related genes in GEPIA 2. TGR5 exhibited moderate positive associations with CHT1 and GPAT1 (R = 0.41, P < 0.05; R = 0.41, P < 0.05), and TGR5 exhibited strong positive associations with CTL1 and IDO1 (R = 0.77, P < 0.05; R = 0.75, P < 0.05). **A** CTL1; **B** CTH1; **C** CHKA; **D** IDO1; **E** GPAT1. *CTL1* transporter-like protein 1, *CTH1* choline transporter 1, *CHKA* choline kinase alpha, *IDO1* indoleamine 2,3-dioxygenase 1, *GPAT1* glycerol-3-phosphate acyltransferase-1

To further validate the potential role of TGR5 in choline metabolism, tryptophan metabolism and glycerophospholipid metabolism, the correlations between TGR5 and these metabolism-related genes in pancreatic cancer were analyzed. The following genes were identified for subsequent validation: the choline metabolism-related genes, choline transporter-like protein 1 (CTL1), choline transporter 1 (CHT1), choline kinase alpha (CHKA); the tryptophan metabolism-related gene indoleamine 2,3-dioxygenase 1 (IDO1) and the glycerophospholipid metabolism-related gene glycerol-3-phosphate acyltransferase-1 (GPAT1) [23–25]. The correlation analyses indicated TGR5 exhibited moderate positive associations with CHT1 and GPAT1 (R = 0.41, P < 0.05; R = 0.41, P < 0.05). Moreover, TGR5 exhibited strong positive associations with CTL1 and IDO1 (R = 0.77, P < 0.05; R = 0.75, P < 0.05) (Fig. 7). These results validated that TGR5 was associated with the choline metabolism, tryptophan metabolism and glycerophospholipid metabolism in pancreatic cancer.

## Discussion

Pancreatic cancer is currently among the most lethal diseases and efficacious therapeutic options are limited [26]. Pancreatic cancer cells adopt metabolic reprogramming to meet the energy demand and result in the carcinogenesis and progression. In addition, metabolic reprogramming is also closely related to chemotherapy, radiotherapy and immunotherapy in pancreatic cancer [27]. Treating cancer by targeting the unique metabolic reprogramming has emerged as a promising therapeutic approach for treatments against pancreatic cancer [28].

Bile acids play an important role in intestinal nutrient absorption, biliary secretion of lipids and toxic metabolites[29]. Moreover, bile acids also participate in regulating energy homeostasis and maintaining metabolic homeostasis as regulatory molecules. Disorders in bile acids synthesis and metabolism may cause metabolic syndrome, inflammation and liver diseases. Bile acids were also identified as a potential carcinogen [2, 30]. Previous studies revealed that bile acids were considered as tumor-promoting factors in the development of human gastrointestinal cancers and elevated levels of bile acids may cause DNA damage [2]. It was also found that bile acids were associated with the risk factors of pancreatic cancer, including alcohol, smoking and a high-fat diet [31, 32]. These risk factors can lead to excess bile acid secretion, which promotes the progression of pancreatitis. Pancreatitis may gradually develop into malignant tumors [33]. Thus, bile acids may be involved in pancreatic cancer carcinogenesis.

TGR5 (also called GPBAR1) is a bile acid-specific cell membrane G protein-coupled receptor linked to cAMP signaling, which participates in bile acids synthesis, energy metabolism and homeostasis. Moreover, previous studies demonstrated that high TGR5 may be a risk factor for cancer, such as cholangiocarcinoma, non-small cell lung cancer (NSCLC). TGR5 overexpression could promote cell proliferation and tumor growth in NSCLC [34–36]. It was found that TGR5 was significantly higher in pancreatic cancer tissues than the adjacent normal tissues. High TGR5 suggested poor survival in pancreatic cancer patients [14]. Our data indicated that the proliferation ability of pancreatic cancer cells was decreased significantly when antagonizing TGR5. Meanwhile, antagonism of TGR5 by SBI-115 induced apoptosis in pancreatic cancer cells. However, the significant alterations in the migratory and invasive abilities of SBI-115-treated pancreatic cancer cells were not observed in our study. It was reported that the levels of cyclic adenosine monophosphate (cAMP) and cell proliferation were decreased in cystic cholangiocytes after treatment of SBI-115. On the contrary, TGR5 agonists increased cAMP levels and the ability of cystic cholangiocytes proliferation [16]. Previous study revealed that blocking cAMP-dependent intracellular signalings may prevent the development of pancreatic cancer. It was also found that the development of pancreatic cancer was prevented by cAMP decrease in hamster models [37, 38]. However, few studies have been reported to directly focus on the role of cAMP in migration and invasion of pancreatic cancer cells.

For further study, the cell morphologies and ultrastructural changes induced by SBI-115 in pancreatic cancer cells were further examined. Morphologically, PANC-1 and BXPC3 cells treated with SBI-115 exhibited unregulated-shaped bodies and more visible vacuoles, and the transmission electron microscopy showed the mitochondria in SBI-115-treated cells exhibited decreased mitochondrial cristae, swollen-shaped body and ruptured mitochondrial membranes. Mitochondria are important organelles for cellular physiology and homeostasis in most eukaryotic organisms, and bile acids could directly act on the mitochondria [39]. TGR5 ablation did influence the mitochondrial biogenetic pathway [40]. Our results demonstrated that antagonism of TGR5 would lead to the abnormal structure of mitochondria in pancreatic cancer cells, but further verification was also still needed.

Meanwhile, the LC–MS-based metabolomics analysis revealed that antagonism of TGR5 could alter the metabolic profiles in PANC-1 cells. A total of 122 differential metabolites were significantly up-regulated and 245 differential metabolites were down-regulated in SBI-115-treated-group when compared with the DMSO-treated group. Furthermore, the KEGG enrichment analyses showed the altered metabolites were significantly related to choline metabolism in cancer, tryptophan metabolism, glycerophospholipid metabolism, et al. The correlation analyses between TGR5 and the metabolism-related genes in GEPIA 2 also suggested that TGR5 was significantly correlated with the choline metabolism, tryptophan metabolism and glycerophospholipid metabolism in pancreatic cancer. Previous studies indicated that choline metabolism, tryptophan metabolism and glycerophospholipid metabolism were altered in a wide variety of cancers. Activated choline metabolism is a hallmark of carcinogenesis and tumor progression, leading to elevated levels of phosphocholine and glycerophosphocholine in multiple cancers [41]. Tryptophan metabolism can promote tumor progression by suppressing antitumour immune responses and increasing the malignant properties of cancer cells [24, 42, 43]. They may be promising targets in the treatment of pancreatic cancer [28, 44].

To the end, there are still some shortcomings in our study. First, more animal models are urgently needed to verify the effect of TGR5 in pancreatic cancer. Second, the basic molecular mechanisms underlying TGR5 in pancreatic cancer have been obscure. Third, SBI-115 is a novel TGR5 antagonist and more exploratory studies are still needed by in vivo animal experiments and in vitro cell experiments. In conclusion, our study demonstrates that antagonism of TGR5 suppresses cell proliferation and induces apoptosis in pancreatic cancer cells. TGR5 may be a potential therapeutic target in pancreatic cancer.

## Supplementary Information

Fig. S1. The KEGG pathway maps of choline metabolism, tryptophan metabolism and glycerophospholipid metabolism. The red and blue nodes represented upregulated metabolites and downregulated metabolites in SBI-115-treated-group, respectively. **A** choline metabolism in cancer; **B** tryptophan metabolism; **C** glycerophospholipid metabolism. KEGG, Kyoto Encyclopedia of Genes and Genomes.

## Data Availability

Derived data supporting the findings of this study are available from the corresponding author on reasonable request.

## Abbreviations

TGR5: Takeda G protein receptor 5
LC-MS: Liquid chromatography mass spectrometry
TEM: Transmission electron microscopy
