# Supplementary figures and images for: Investigation of the potential role of TGR5 in pancreatic cancer by a comprehensive molecular experiments and the liquid chromatography mass spectrometry (LC–MS) based metabolomics
Source: Discov Oncol. 2022 Jun 11;13:46. doi: 10.1007/s12672-022-00504-2 (PMC9188013; doi:10.1007/s12672-022-00504-2)

A


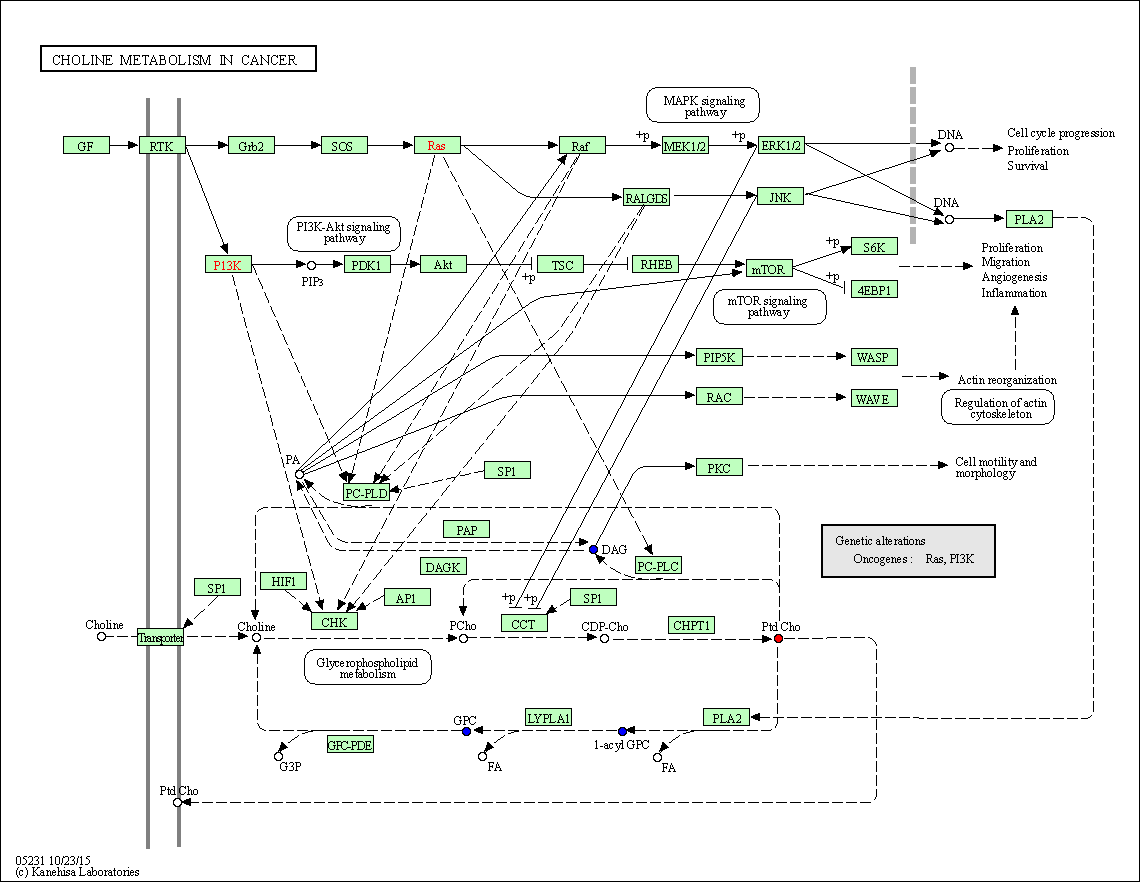


B


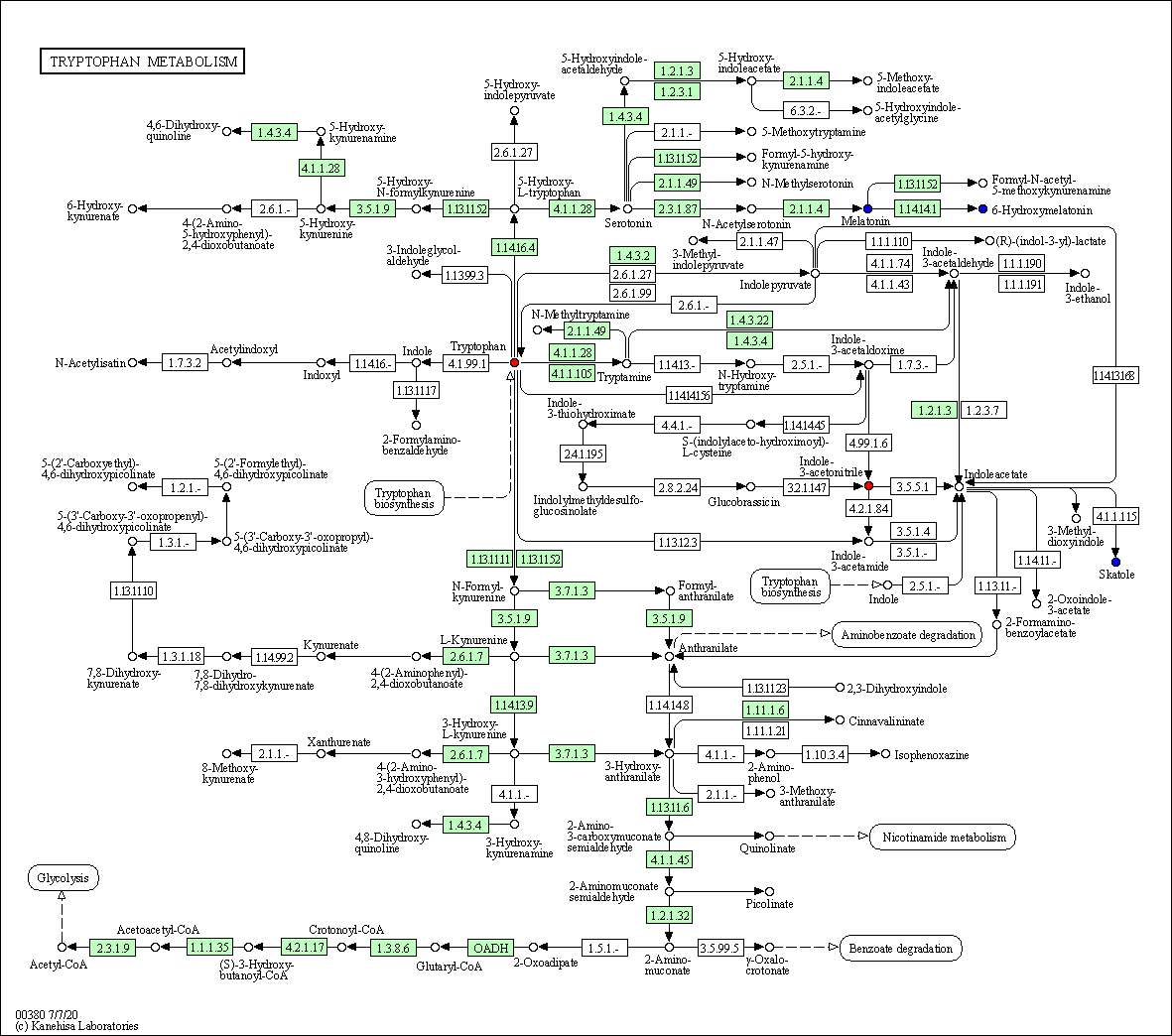


C


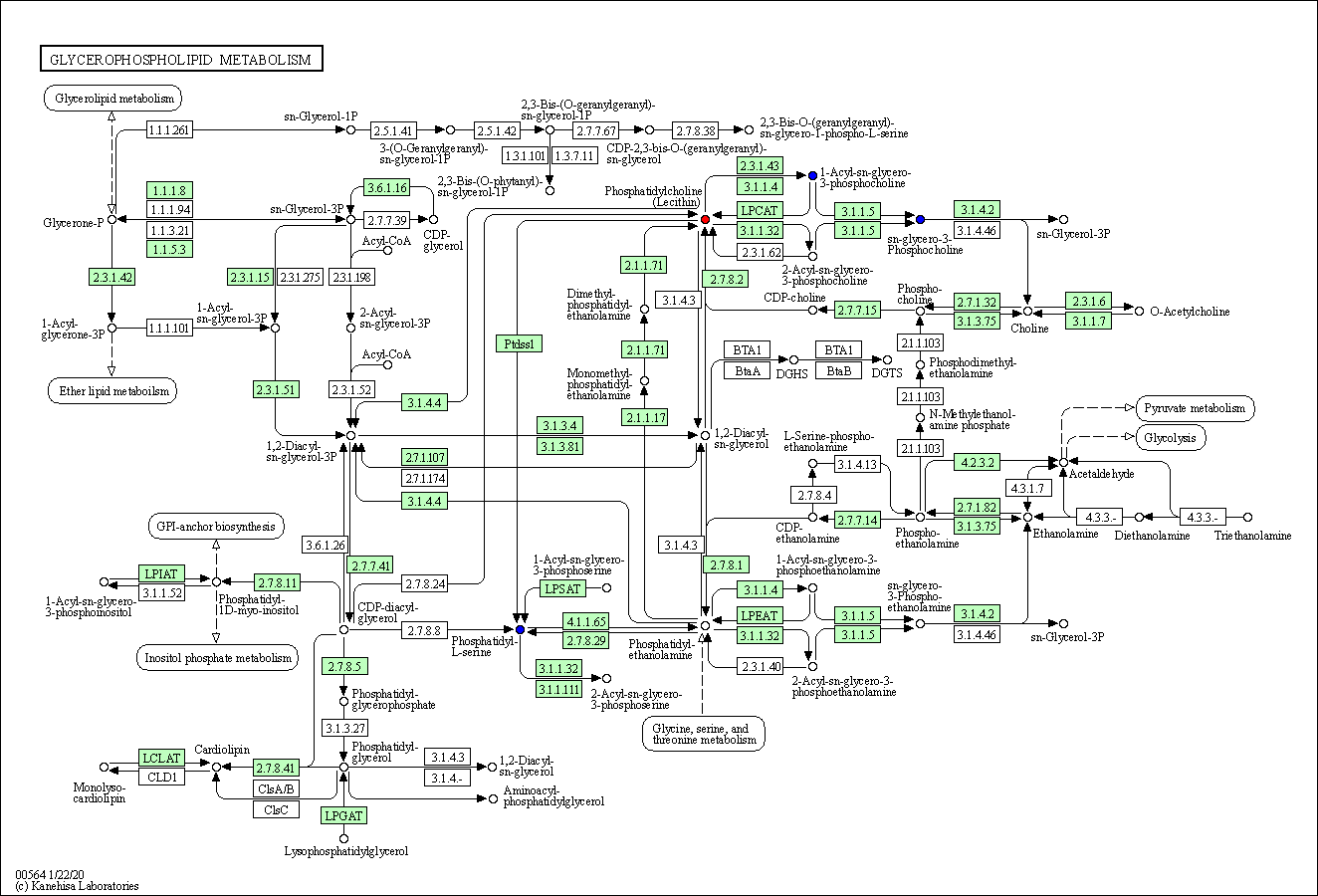

Supplement: Supplementary file 1 — Fig. S1. The KEGG pathway maps of choline metabolism, tryptophan metabolism and glycerophospholipid metabolism. The red and blue nodes represented upregulated metabolites and downregulated metabolites in SBI-115-treated-group, respectively. A choline metabolism in cancer; B tryptophan metabolism; C glycerophospholipid metabolism. KEGG, Kyoto Encyclopedia of Genes and Genomes. [file 12672_2022_504_MOESM1_ESM.docx]
